# Signs of Intracranial Hypertension, Hypermobility, and Craniocervical Obstructions in Patients With Myalgic Encephalomyelitis/Chronic Fatigue Syndrome

**DOI:** 10.3389/fneur.2020.00828

**Published:** 2020-08-28

**Authors:** Björn Bragée, Anastasios Michos, Brandon Drum, Mikael Fahlgren, Robert Szulkin, Bo C. Bertilson

**Affiliations:** ^1^Division of Family Medicine and Primary Care, Department of Neurobiology, Care Sciences and Society, Karolinska Institutet, Solna, Sweden; ^2^ME-center, Bragée Clinics, Stockholm, Sweden; ^3^Academic Primary Health Care Center, Stockholm Health Care Services, Region Stockholm, Stockholm, Sweden

**Keywords:** fatigue syndrome, chronic, pain, Ehlers Danlos syndrome, Arnold-Chiari malformation, magnetic resonance imaging, intracranial hypertension, pseudotumor cerebri, hypermobility, joint

## Abstract

The pathophysiology of myalgic encephalomyelitis/chronic fatigue syndrome (ME/CFS) is unknown. In this study, we test the hypothesis that hypermobility, signs of intracranial hypertension (IH), and craniocervical obstructions may be overrepresented in patients with ME/CFS and thereby explain many of the symptoms. Our study is a retrospective, cross-sectional study, performed at a specialist clinic for referred patients with severe ME/CFS as defined by the Canada Consensus Criteria. The first 272 patients with ME/CFS were invited to participate, and 229 who provided prompt informed consent were included. Hypermobility was assessed using the Beighton Score. IH was assessed indirectly by the quotient of the optic nerve sheet diameter (ONSD)/eyeball transverse diameter on both sides as measured on magnetic resonance imaging (MRI) of the brain. We also included assessment of cerebellar tonsil position in relation to the McRae line, indicating foramen magnum. Craniocervical obstructions were assessed on MRI of the cervical spine. Allodynia was assessed by quantitative sensory testing (QST) for pain in the 18 areas indicative of fibromyalgia syndrome (FMS). A total of 190 women, mean age 45 years, and 39 males, mean age 44 years, were included. Hypermobility was identified in 115 (50%) participants. MRI of the brain was performed on 205 participants of whom 112 (55%) had an increased ONSD and 171 (83%) had signs of possible IH, including 65 (32%) who had values indicating more severe states of IH. Cerebellar tonsils protruding under the McRae line into the foramen magnum were identified in 115 (56%) of the participants. MRI of the cervical spine was performed on 125 participants of whom 100 (80%) had craniocervical obstructions. Pain at harmless pressure, allodynia, was found in 96% of the participants, and FMS was present in 173 participants or 76%. Compared to a general population, we found a large overrepresentation of hypermobility, signs of IH, and craniocervical obstructions. Our hypothesis was strengthened for future studies on the possible relation between ME/CFS symptoms and hypermobility, IH, and craniocervical obstructions in a portion of patients with ME/CFS. If our findings are confirmed, new diagnostic and therapeutic approaches to this widespread neurological syndrome should be considered.

## Introduction

Myalgic encephalomyelitis/chronic fatigue syndrome (ME/CFS) is characterized by severe unmitigable fatigue, post-exertional malaise (PEM), pain, and neurological and immunological dysfunction as noted in the Canada Consensus Criteria (CCC) for ME/CFS from 2003 (1).

The true prevalence of ME/CFS is unknown although previous international studies provide estimates of 0.2 to 1.6% (2). ME/CFS is more common in women aged 30–39 years. The disorder results in large costs of treating and managing this syndrome, which are estimated to exceed $20 billion annually in United States alone (3). The pathophysiological origins of ME/CFS remain unclear. Since the 1960s, a multitude of hypotheses of causality have been proposed, most of which explain disease origins with an infection. A number of bacteria and viruses have been proposed as ME/CFS-causative although there remains no broad consensus (4). The World Health Organization now classifies ME/CFS as a neurological disease of postviral origin (G93.3) (5).

At the request of the Stockholm Region County Council, we started an ME/CFS specialty clinic in 2017 aimed at ME/CFS diagnosis, treatment, and research. Using an extensive neurological protocol, we found that patients frequently had clinical findings including hypermobility and central nervous system (CNS) pathologies, including magnetic resonance imaging (MRI) findings in the brain and the craniocervical region. Thus, we hypothesize that hypermobility and craniocervical obstruction are overrepresented in patients with ME/CFS and that a large portion of these patients may have a degree of intracranial hypertension (IH), which may explain many of the ME/CFS symptoms. To our knowledge, this is the first study of ME/CFS focusing on a possible mechanical pathophysiology.

## Methods

### Study Design and Participants

This study is a retrospective cross-sectional study of consecutive patients referred to our open care specialty ME/CFS clinic. Ethical approval was granted by the Swedish Ethical Review Authority (nr. 2019-01566).

At the start of the study, 620 patients had been referred to our clinic, of whom 272 were diagnosed with ME according to the CCC (ICD G93.3). Each of these patients was asked via a letter to participate in the study, and 229 who provided informed consent to participate within 6 weeks were included. Data were extracted from patients' medical records.

### Hypermobility and Neurological Assessment

Hypermobility was assessed using nine tests to create a Beighton Score (from 0 to 9) with five or more indicating hypermobility (6). General joint hypermobility according to cutoff values stated by Singh was identified (7). Neurological assessment was done using discomfort drawings and quantitative sensory testing (QST). Discomfort drawings were assessed for pattern of neuropathic pain and pattern of widespread pain (7). QST was performed using an electronic device, the Somedic® algometer, to establish the threshold for skin pressure pain. The pressure was gradually increased with a 1 cm^2^ surface area probe at stipulated areas in all four body quadrants. The pain thresholds for pressure were measured at 18 locations according to the 1990 ACR protocol, and participants with 11 or more stipulated areas with pain threshold values under the normal 400 kPa/cm^2^ met criteria for fibromyalgia syndrome (FMS) (8). The number of pain threshold measurements from 1 to 399 kPa/cm^2^ and from 1 to 199 kPa/cm^2^ were recorded. The criteria for FMS was evaluated for all participants and required tenderness on pressure (tender points) in at least 11 of 18 specified sites and the presence of widespread pain for more than 6 months for diagnosis. Widespread pain is defined as axial pain, left- and right-sided pain, and upper and lower segment pain.

### MRI of the Brain

MRI scans of the brain were offered to all participants. MRIs were conducted in different labs and included T1- or T2-weighted scans with or without fluid-attenuated inversion recovery (FLAIR) with a section thickness of 3 mm. Scans were assessed by an experienced radiologist through the Regions MRI database, using Sectra® software, which offers built-in measurement tools. An interrater reliability (IRR) study of the MRI assessments was performed on 100 randomized participants for whom the radiologist assessments were compared to those made by a resident physician and a medical student.

**Figure 1:**
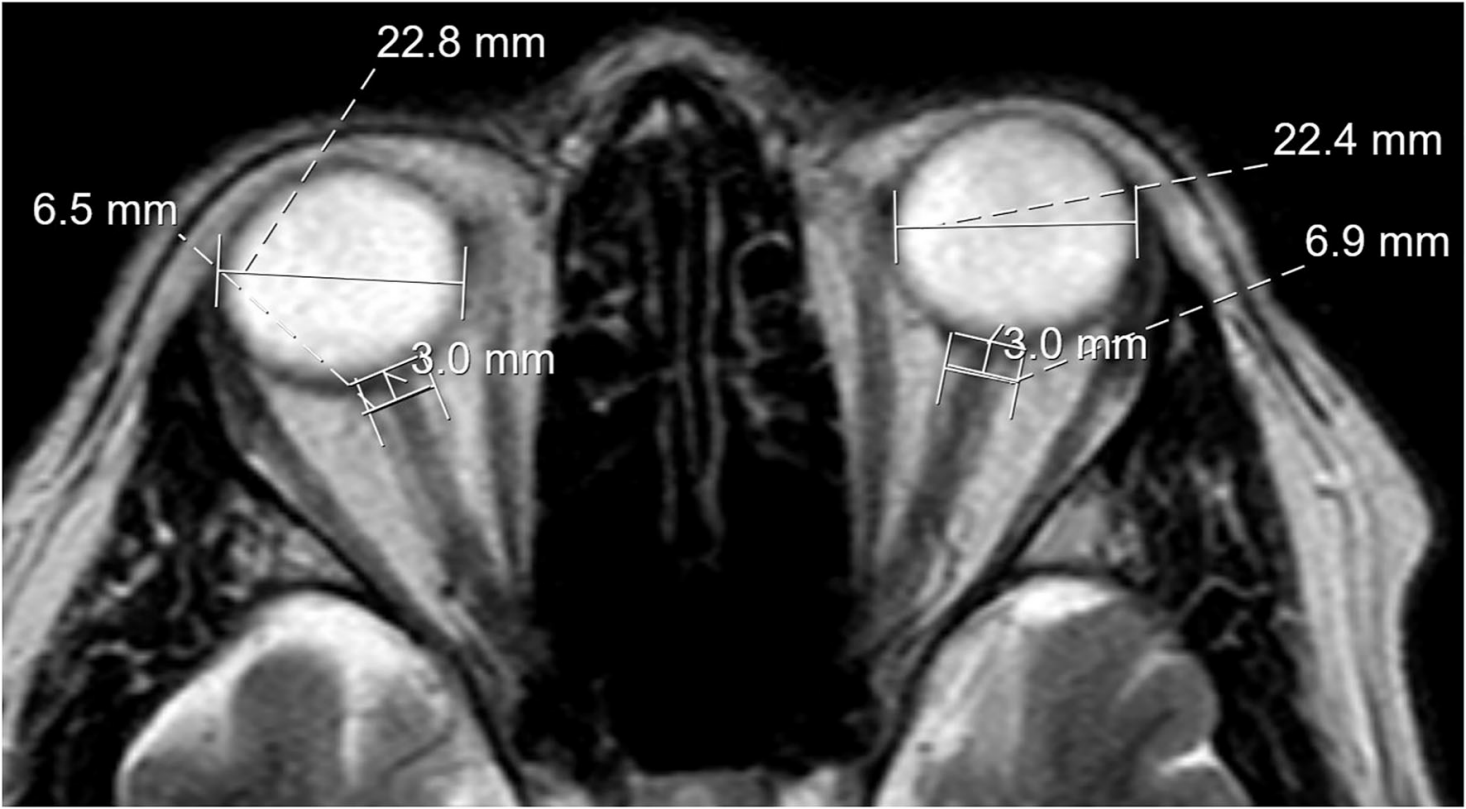
MRI scan demonstrating eyeball transverse diameter and optic nerve sheath diameter measurements, which, in this case, indicates abnormal widening.

The eyeball transverse diameter (ETD) was measured on axial sections of T2-weighted images. This was measured in the posterior chamber at the largest diameter of the retina's inner edges from left to right. The optic nerve sheath diameter (ONSD) was measured on the same axial T2 sections 3 mm from the midline of the optic nerve's inner limit to the posterior chamber along its axis. If the measurement did not allow a precision of 1/10 mm due to low resolution, the distance was appreciated to 1/10 mm from the external margins of the thick optic sheath layers covering the optic nerve (Figure 1). The range for normal ONSD is 4.8–5.8 mm, and a value of >5.8 mm corresponds to an elevated CSF pressure (>25 mm Hg) indicating IH (9). The ONSD/ETD ratio has a normal value of 0.19 ± 0.02, and values > 0.25 are related to IH with severe symptoms (10, 11).

**Figure 2:**
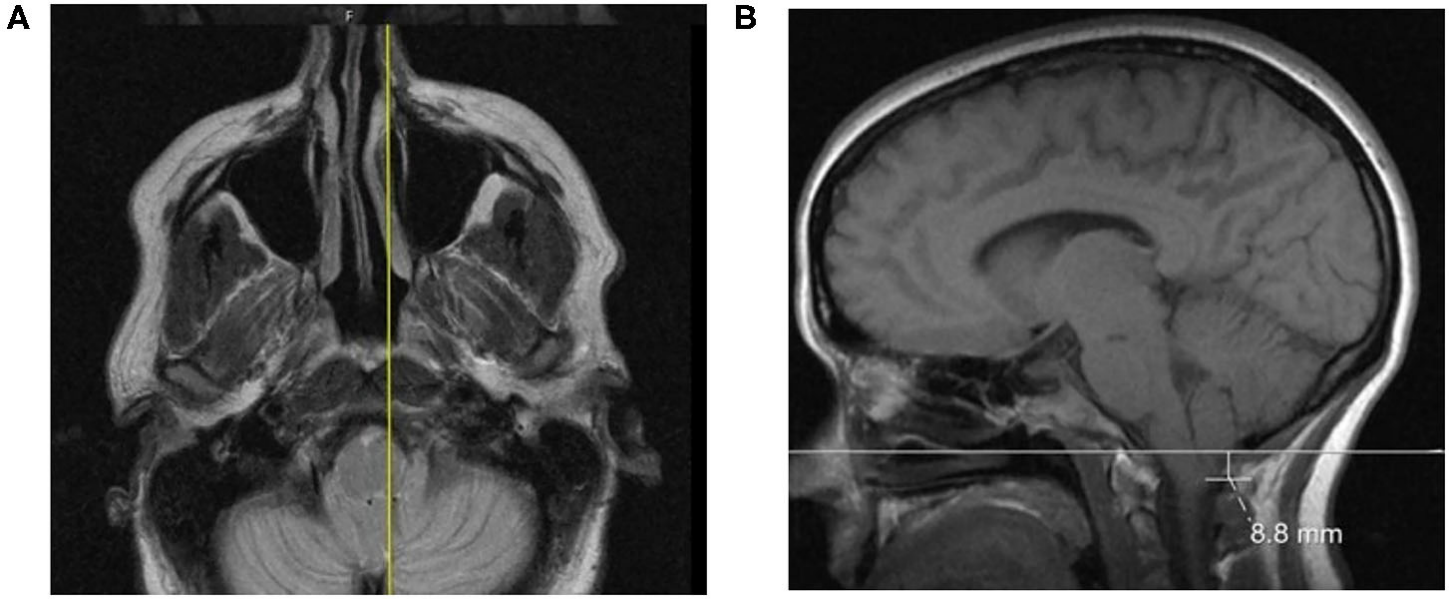
**(A)** A case featuring a right-sided, low-lying cerebellar tonsil; **(B)** The axial projection position.

The foramen magnum was identified and measured on a midsagittal slice on T1-weighted scans. Distances were measured by the length of a line (McRae line) drawn on a sagittal skull radiograph joining the basion and opisthion as described by FW Smith (Figure 2A) (9). The position of the cerebellar tonsils and, if so, degree of herniation were measured as the distance from the McRae line to the most inferior point of the tonsil on frontal or sagittal projections on T1- or T2-weighted MRI scans (Figure 2B).

The clivo-axial angle (CXA) was measured on a midline sagittal slice on T1-weighted scans. A straight line was drawn along the caudal edge of the clivus bone, and a second was drawn by extending a straight line along the posterior axial line from the base of the axis to the dens. The angle at the intersection of these two lines was then measured. A CXA of <150° was considered abnormal as stated by Henderson et al., who in 2018 reported a growing recognition of the relationship between a kyphotic CXA and the risk of brainstem deformity and craniocervical instability (10).

All variables were recorded and rounded to the nearest millimeter except the CXA, which was measured in degrees.

### MRI of the Cervical Spine

When clinical findings were suggestive of cervical spine pathology, an MRI from C1 to T2 was ordered in cases lacking recent imaging. MRI scans of the cervical spine were acquired and assessed using the same labs and machines as used for MRI of the brain. Spondylolisthesis (forward or backward slipping of a vertebral body), osteophytes (bone formations that may obstruct nerve and other soft tissue passages), spinal cysts, and syringomyelia were noted regardless of segment or severity. Spinal cord compression due to bulging or herniated discs or bones impinging upon the medulla spinalis was noted on axial and sagittal sections. The level of most prominent reduction of space in the spinal cord was noted as well as the degree of reduction. The area immediately above and below the most reduced area was compared to the area in which the most prominent reduction was located. All areas were measured in mm^2^, and the difference was then calculated as a percentage of reduction compared with the closest normal segments. Correlation between the degree of joint hypermobility and the ONSD/ETD ratio as a marker for IH was calculated.

## Results

### Characteristics of the Participants

There was a skewed sex distribution among the participants with 83% being women. The mean age of the 190 female participants was 45 years and, for the 39 men, 44 years (range 17–75 years). The mean body mass index (BMI) of participants was 25.1 for women and 24.9 for men. A BMI > 25 was noted in 101 (44%) participants, and a BMI > 30 was noted in 34 participants (15%).

The mean duration of ME-related symptoms was approximately 9 ± 8 years. We could assess earlier care contacts through our regional common medical record system for 198 participants. The mean number of contacts with the health care system during the last 5 years was 90. The mean number of diagnoses that participants had received over the last 5 years included 20 somatic diagnoses and three psychiatric diagnoses. A total of 150 (76%) participants had received at least one psychiatric diagnosis. The diagnosis of FMS had been given to 31 participants (16%). There were 48 participants (25%) who had received an ME/CFS diagnosis before first admission to the clinic.

The prevalence of permanent sick leave was 39% and, together with other forms of social welfare, 57% of the participants had their income covered by social insurance, 10% had no income at all, and 25% still worked to some degree. The education level was high as 40% of participants had previous or actual work with an academic background, and 10% had blue-collar work.

### Hypermobility and Neurological Assessments

General joint hypermobility was identified in 115 participants or 50% (Table 1). A total of 93 participants (41%) had Beighton scores of >4 points. A diagnosis of hEDS when criteria other than hypermobility are considered had been given earlier or after admission to 44 participants (20%).

**Table 1:** Prevalence of general hypermobility in different groups with cutoff values according to Singh (7).

**Age**	**Sex**	**Cutoff value**	**Number of participants**	**Of whom with GJH**	**Prevalence (%)**
14–19	Male	≥4	2	2	100%
	Female	≥5	2	1	50%
20–39	Male	≥4	10	5	50%
	Female	≥4	65	36	55%
40–59	Male	≥2	21	9	43%
	Female	≥4	103	54	52%
60–69	Male	≥1	6	2	33%
	Female	≥3	16	5	31%
70–101	Female	≥2	4	1	25%
Sum			229	115	50%

Assessment of pain drawings could be made in all but four participants, and 192 or 85% marked pain in all four body quadrants—that is, widespread pain.

Allodynia criteria, defined as pain at harmless pressure, were met in 219 participants (96%) with 153 (67%) having >11 painful areas with a limit of 200 kpa/cm^2^, that is, half the criteria limit for FMS. The criteria for FMS were met in 173 of our 229 participants, that is, in 76%.

### MRI of the Brain

MRI exams of the brain were performed on 205 (90%) participants. Twenty-four participants did not undergo a brain MRI scan due to claustrophobia or other contraindications. The exams were performed within 6 months before or after the first visit to the clinic in 171 participants (84%). Examiner IRRs ranged between 0.77 and 0.88 for the assessment of ONSD, ETD, tonsil position, and craniocervical obstructions.

ONSD had a mean of 5.64 ± 0.67, and values >5.8 mm were found bilaterally in 61 participants and unilaterally in 51 participants, that is, all together in 55% of the participants. ETD had a mean value of 23.5 ± 1.1 mm bilaterally. The mean ONSD/ETD was 0.24 ± 0.03 and a value >0.22 on either side was found in 171 of our participants (83%). An ONSD/ETD larger than 0.25 was identified in 87 participants (42%).

The position of the cerebellar tonsils under the McRae line of the cerebellar tonsils had a mean value of 0.80 ± 2.95 mm. Negative values indicate a position above the McRae line, and positive values represent a position below the line (Table 2).

**Table 2:** The position of the cerebellar tonsils in relation to the McRae line as measured on MRI scans of the brain in participants with ME/CFS (*n* = 205) and in the general population.

	**Number of participants**	**Study prevalence (%)**	**Normal prevalence (%)**	**Comments (overrepresentation)**
A Both tonsils >2 mm above line	22	11	50	No obstruction (−5X)
B Both tonsils in line or 1–2 mm above line	68	33	25	No obstruction (=)
C Any tonsil under line	115	56	25	Obstruction (2X)
D Any tonsil 3–4 mm under line	51	25	8	Tonsillar ectopia (3X)
E Any tonsil 5 mm or more under line	27	13	3	Chiari 1 (4X)
F Both tonsils 5 mm or more under line	7	3	1	Chiari 1 (3X)

*A participant could be included in two groups (C-E). Normal prevalence was determined by Smith (A–E) (12) Vernooij (F) (13)*.

The mean CXA was 148° ± 10°, and 114 participants (56%) had a CXA of <150°.

### MRI of the Cervical Spine

An MRI scan of the cervical spine was or had been performed in 125 participants (55%).

Spondylolisthesis was identified in eight (6%) and osteophytes in 11 (9%) participants. Spinal cysts or syrinxes were not identified in any scan. Obstructions of different varieties were present in 100 participants (80%). More than one segment of C1–T2 was obstructed in 80 participants (64%). Spinal cord compressions were most frequent at C5–C6 (53%) and C6–C7 (28%). The age distribution and findings of obstructions is presented in Table 3.

**Table 3:** Findings of obstructions in the cervical spine on MRI scan of the cervical spine. Number of participants.

**Age**	**All participants**	**MRI exams**	**Obstr. findings**	**% of MRI exams**
18–19	2	1	0	0
20-29	30	15	7	47
30–39	47	28	21	75
40–49	72	39	32	82
50–59	52	30	28	93
60–69	22	10	10	100
70–75	4	2	2	100
All	229	125	100	80

## Discussion

Our hypothesis that general joint hypermobility and craniocervical obstructions is overrepresented in patients with ME/CFS and that many of these patients may have a degree of IH was supported by our findings outlined above. Based on these findings, we propose that joint hypermobility and craniocervical obstructions may be one pathway to develop ME/CFS. ME/CFS as defined by the large CCC umbrella may include subgroups with infectious, immunological, traumatic, and craniocervical origins. The complexity of ME/CFS and the difficulty faced in diagnosing this syndrome is reflected by the numerous healthcare contacts with 90 visits in the last 5 years although the mean in the general population, including those with chronic diseases, is <30. The many diagnoses our participants had encountered prior to admission at our clinic is also conspicuous.

Joint hypermobility was overrepresented in our study as 49% of the participants with ME/CFS had a Beighton score >4 compared to 3% in the general population (6). To discriminate between ME/CFS and Ehlers Danlos syndrome hypermobility type (hEDS), other features were also considered, including overly relaxed skin, a history of joint displacements, bruising, and a family history including or typical for hEDS. A Beighton score cutoff value for hEDS is 5 or more. The diagnosis of hEDS was made or confirmed by the physicians at the clinic, specialists who are experienced in pain medicine. Unfortunately, the genetic clinic in the region does not accept referrals for hEDS without suspicion of the vascular type of EDS, and there is no available specific biomarker to confirm the diagnosis of hEDS. A diagnosis of hEDS was present in 20% of our study population, and the prevalence in the general population is <1% (11). Such an overrepresentation of connective tissue disorders, such as hEDS, has, to the best of our knowledge, not been previously described in a large adult ME/CFS cohort. However, the comorbidity between hEDS and ME/CFS has been proposed in many reviews and shown in a cohort of 68 children and controls previously (14). Furthermore, numerous neurological and spinal manifestations of hEDS are known, giving symptoms similar to those in ME/CFS (15).

ONSD and the ONSD/ETD ratios are related to IH. Our results indicate that IH may contribute to ME/CFS symptoms (Table 4). ONSD ratios indicating IH were significantly more common in our study cohort compared to that of the healthy adult population as described in a study by Kim et al. of 314 individuals (16). ONSD values of >5.8 mm, indicating IH, were found in a majority of our participants. However, when we calculated ONSD/ETD ratios, which are considered a more adequate predictor of IH than ONSD as they eliminate body-size-related variability, 171 participants (83%) had ratios >0.22. ONSD/ETD ratios >0.22 has been found in 5% of the normal population (16). The ratio has a small variation as the normal value is 0.19 ± 0.02. In our population, 65 participants (32%) had an ONSD/ETD ratio >0.25, indicating a considerable and significant difference. In a study of 1,766 participants with either IH or intracranial hypotension, an ONSD/ETD ratio of 0.29 ± 0.04 was used as an indicator of significantly elevated intracranial pressure (17) although 0.25 was correlated to IH in a separate study of patients who underwent surgery for vascular infarctions (18). This suggests that a portion of patients with ME/CFS may have more harmful intracranial pressure.

**Table 4:** A comparison of our study population (*n* = 205) and a healthy reference population (*n* = 314) for optic nerve sheath diameter and eyeball transverse diameter as assessed by MRI scan of the brain.

	**Bragée ME-center (±SD)**	**Reference population (±SD) (16)**	*p*-value^*^
ONSD mm	5.64 ± 0.67	4.71 ± 0.31	<0.001
ETD mm	23.47 ± 1.05	21.24 ± 0.79	<0.001
ONSD/ETD ratio	0.24 ± 0.03	0.22 ± 0.01	<0.001

* *Per two sample t-test using the published means from reference population. ONSD, Optic Nerve Sheath Diameter; ETD, Eyeball Transverse Diameter*.

Low cerebellar tonsils that protrude into the foramen magnum may obstruct the flow of CSF and indirectly cause IH. The limit for what is considered a low position of cerebellar tonsils varies between investigators, and most argue that a position >5 mm below the McRae line bilaterally should be considered a Chiari 1 malformation. That criteria was fulfilled by 3.4% of our participants as compared to prevalence in a normal population, which is estimated to be 0.3–1%, indicating at least a three-fold higher prevalence in our participants with ME/CFS (12). Others have used a definition for Chiari 1 malformation when the position of the tonsils are >3 mm below the McRae line, which occurred in 4% of patients referred for MRI for different diagnosis in a retrospective study of 2,480 MRI scans (19). Normal values vary with age and sex, and the position of the cerebellar tonsils rises with age due to general cerebral atrophy (12). Only a fraction (11%) of our participants with ME/CFS had a normal cerebellar tonsil position well above the McRae line. Symptom onset in our study population was greater in the 25–45 age range, when symptoms of Chiari syndrome usually also first present (20).

Interestingly, a high proportion of our participants with ME/CFS exhibited signs of IH, and the criteria for ME/CFS include many symptoms similar to those of IH, including cognitive dysfunction, headache, dizziness, and pain. We contend that IH symptoms should not exclude patients from being evaluated for and potentially diagnosed with ME/CFS. A connection between ME/CFS and IH has previously been suggested by Higgins 2017 (21). In Higgins' study, 5 out of 20 patients with ME/CFS also had increased CSF pressure, >20 cm H_2_O.

Also interesting is that 173 or 76% of participants had concomitant FMS. The prevalence of allodynia was even higher at 96%. This finding, characteristic of widespread sensitization, is a strong argument for CNS engagement in ME/CFS. A shared pathophysiology between ME/CFS, IH, and FMS has also been hypothesized by Hulens (22). Other criteria for FMS are illness of more than 3 months and pain in all four body quadrants. As all participants had a pain duration of more than 6 months, which is a criterion for referral to the clinic, and the majority also fulfilled the criteria of pain distribution, FMS seems to be a very common comorbidity even if that diagnosis only had been given to 25% of the participants prior to admission.

The correlation between the degree of joint hypermobility and the ONSD/ETD ratio as a marker for IH was very weak. There might be unknown confounders, or both IH and joint hypermobility can contribute to ME/CFS symptomatology independently.

Craniocervical obstructions were frequent in our study sample. Most of these were disc bulges and hernias, which were found in 80% of participants who underwent MRI of the cervical spine. These kinds of obstructions are increasingly frequent with increased age and may be asymptomatic. However, our study population was relatively young with a mean age of 45 years. One of the surprisingly few studies on the normal prevalence of craniocervical obstructions is the still often cited work by Boden from 1990, in which 63 asymptomatic volunteers participated (23). Of the 40 participants with age <40 years, four participants or 10% had disc bulging or hernias; of the 23 participants with age >40 years, two participants or 9% had such findings. If we presume that all of our participants without a present MRI scan are free from findings of obstruction of this kind (which gives an underestimate of the true obstruction prevalence in our sample), we still find a significant overrepresentation compared with the groups in Boden's study. The prevalence of cervical hernias or disc bulging in our study with this presumption is 35% with such findings in 28 of altogether 79 participants <40 years of age. In the group >40 years of age, we find such obstructions in 48%, that is, in 72 of altogether 150 participants. Comparing these prevalence's with Boden's study using a Chi-square test shows a significant overrepresentation of craniocervical obstructions in our study, both in the age group <40 years (*p* = 0.003) and in those >40 years (*p* = 0.004).

In the Wakayama Spine Study, a more recent study on spine pathology from 2014, Teraguchi and others studied the prevalence of spine disc degeneration grade 3–4 in the whole spine of 975 Japanese volunteers from the general population (24). The findings in that study correspond well to the craniocervical obstructions we observe as disc bulging or disc hernia. The Wakayama cohort is interesting as also patients with different symptoms were recruited, for example, 25% reported neck pain. It should, therefore, reflect the general population rather than the healthy population. This Wakayama Spine Study makes it possible to also compare prevalence of craniocervical obstructions in both males and females. In ME/CFS, there is an overrepresentation of women, and the relatively small number of men in our study does not justify proper statistical evaluation of differences in prevalence. However, for females and the cohort as a whole, we found a significantly higher proportion of craniocervical obstructions, in particular, among females younger than 50 years of age (*p* < 0.01). In our cohort, 25 participants had no cervical obstructions and 100 had. In the Wakayama study, 367 participants had no cervical obstructions, and 608 had. The difference is significant with *p* < 0.01 using Fisher exact test. See Table 5.

**Table 5:** MRI findings of craniocervical obstructions with comparison to the Wakayama spine study by Teraguchi et al. (24).

	**Female participants (number of)**					**Male participants (number of)**				
	**Our study**		**Wakayama study**			**Our study**		**Wakayama study**		
**Age**	**No obstr**.	**Obstr**.	**No obstr**	**Obstr**.	^*^Stat.diff.	**No obstr**.	**Obstr**.	**n:o**	**No obstr**.	**Stat. diff**
<50	18	57	63	24	*p* < 0.01	5	3	28	10	n.s
50–59	1	24	59	57	*p* < 0.01	1	4	31	28	n.s
60–69	0	7	72	86	*P* < 0.05	0	3	22	43	n.s
70–79	0	2	48	124	n.s	0	0	17	72	n.s
>79	0	0	17	101	n.s.	0	0	10	63	n.s.
All	19	90	259	392	*p* < 0.01	6	10	108	216	n.s

* *Statistical difference between groups using Fischer exact test*.

Craniocervical obstructions causing changed CSF flow and neuronal dysfunction have been proposed as a possible vehicle to develop ME/CFS symptoms. In a recent study from Johns Hopkins University, three patients with cervical spinal stenosis and ME/CFS who underwent decompressive spine surgery were considerably improved and relieved from ME/CFS symptoms (25). Another common observation in patients with ME/CFS is that they find relief from nausea, vertigo, and pain symptoms in a supine position. This observation prompts the hypothesis that an upright position may alter CSF and blood flow in the craniocervical area due to the weight of the head compressing cervical segments and gravity pulling cerebellar tonsils caudally/into the foramen magnum. This hypothesis is supported by a pilot study by Freeman et al. on 1,200 patients with neck pain in which they found that both cervical spine obstructions and tonsil position were more prominent in MRI exams conducted in the upright position (9). Consequently, in a future study, patients with ME/CFS should be examined with upright MRI of the craniocervical area and compared with the standard supine MRI.

Several limitations may be noted in our study. First, our patient cohort may differ from what is seen in other clinics as referral to our clinic requires that patients have severe symptoms indicating ME/CFS. Second, the CCC are inclusive and may not discriminate sufficiently between other disorders, such as idiopathic intracranial hypertension (26). Third, MRI assessments were done by a single radiologist and, thus, not confirmed. However, we made an IRR assessment between the radiologist, a resident physician, and a medical student, and we found good-to-excellent reliability for most assessments. Fourth, we did not perform a direct measurement of IH as this would have required the use of invasive methods. However, measuring ONSD is considered a reliable method for indirectly measuring IH and corresponds to directly measured intracranial pressure (17).

Neuroinflammation of the CNS is a proposed consequence of craniocervical obstructions and IH. This idea was recently raised by articles on ME/CFS and IH (24, 25). Other mechanisms that may cause neuroinflammation in the CNS are also possible. For instance, the “glymphatic system,” a functional metabolic waste clearance system that engages the CNS, can contribute to IH, and obstructions or flow disturbances can compromise the glymphatic system (27). Komaroff recently expressed this view of ME/CFS pathophysiology (3). We have no ground to say that cervical obstructions cause neuroinflammation and ME/CFS; however, our observation that craniocervical obstructions were very frequent in our population of patients with ME/CFS prompts a question: Is there a substantial subgroup of patients, worldwide, with ME/CFS for whom findings of craniocervical obstructions are signs of undetected IH?

Findings and symptoms from the head and neck region, shoulder, and arms should not be seen only as common complaints among patients with ME/CFS. Rather, as relevant information for future studies to evaluate a possible correlation between craniocervical obstructions, IH, ME/CFS, and neuroinflammation of the CNS. Future studies may also consider a common pathological pathway between ME/CFS and FMS.

## Conclusions

In this relatively large novel study on symptoms and signs of IH, hypermobility and craniocervical obstructions in patients with ME/CFS we found to have a significant overrepresentation in our cohort compared to the general population. These signs might explain some of the major clinical symptoms and signs of ME/CFS, such as brain fog, fatigue, orthostatic intolerance, PEM, preference for the supine position, widespread pain, CNS neuroinflammation, immunological reactivity, and autoimmunity mechanisms. If our findings are further validated, a paradigm shift in the diagnostic methods and treatments for patients with ME/CFS may occur.

## Data Availability Statement

The data from the study are available from the publication date in a de-identified form to other investigators whose proposed use of the data has been approved by an independent review committee and after proposal approval, but should not be spread or used for purposes beyond data confirmation.

## Ethics Statement

The studies involving human participants were reviewed and approved by Swedish Ethical Review Authority (nr. 2019-01566). The patients/participants provided their written informed consent to participate in this study.

## Author Contributions

Conceptualization was made by BBr and BBe. Methodology was created by BBr, software usage by BBr, validation of results by AM, MF, and BD. Formal analyses were completed by BBr. and RS, investigations made by BBr, AM, MF, and BD, resources arranged by BBr Data curation was performed by BBr. The manuscript was written by BBr and reviewed and edited by BBe. Data were visualized by BD and BB. The project administrator was BBe and BBr was responsible for funding acquisition. All authors contributed to the article and approved the submitted version.

## Conflict of Interest

The authors declare that the research was conducted in the absence of any commercial or financial relationships that could be construed as a potential conflict of interest.
